# Associations between the infant gut microbiota and the living environment

**DOI:** 10.1080/29933935.2025.2603823

**Published:** 2025-12-31

**Authors:** Brandon Hickman, Anne Salonen, Kaija-Leena Kolho, Willem M. De Vos, Katri Korpela

**Affiliations:** a Human Microbiome Research Program, Faculty of Medicine, University of Helsinki, Helsinki, Finland; b New Children's Hospital, Pediatric Research Center, Helsinki University Hospital and University of Helsinki, Helsinki, Finland; c Laboratory of Microbiology, Wageningen University, Wageningen, The Netherlands; d Department of Bacteriology and Immunology, University of Helsinki, Helsinki, Finland

**Keywords:** Infant gut microbiota, environment, air pollution, land use

## Abstract

The human gut microbiota is central to the health and development of the host, and the early-life microbiota is affected by a range of factors that can alter the infant's development for years to come. The role of the external, natural environment in shaping the gut microbiota is still largely unknown. We examined how the environment surrounding the home postnatally is associated with the infant gut microbiota in the first 2 y of life. The study utilized 16 s rRNA data from 893 children's fecal samples from the longitudinal birth cohort HELMi. We show that the environment has a minimal overall association with microbiota development (R^2^ < 1%). Air quality explained the greatest degree of variation in microbiota composition, while only forests, agriculture and inland wetlands near the home had any significant association with bacterial genera. The results suggest that the infant gut microbiota is not strongly dependent on the external natural environment, and that the impact of the environment is mostly due to exposure to air pollution that may affect the host's immune system and indirectly the gut microbiota.

## Introduction

The gut microbiota plays an important role in health and development in early life. Colonization of the infant gut begins at birth through exposure to maternal fecal bacteria,^1^^,^^2^ but a number of factors affect the development of the gut microbiota throughout the first years of life, such as antibiotics,^3^^,^^4^ probiotics,^5^ diet,^6^^,^^7^ and birthmode.^8^^,^^9^ The role of environmental exposures on the infant gut microbiota, however, is poorly known.

Experiments designed to test the importance of environmental microbiota exposure have shown an increase in gut and skin microbial diversity when subjects were exposed to soil- and plant-based material.^10-12^ However, it should be noted that the microbial sources of the tested materials included also animal and human fecal matter in addition to any environmental microbes. A study investigating nature exposure and host-associated microbiota in adults did not see any changes,^13^ although this study was limited by the number of subjects. Many studies assessing the role of urbanization in gut microbiota are comparative studies of populations in rural and urban areas. Studies have reported largely on how urbanization, in terms of changes to lifestyle and diet, are associated with the gut microbiota and do not differentiate the environment from these covariates.^14^ While others do not adequately adjust for lifestyle, diet or medical differences between the study groups.^15^ Several studies have observed significant differences in skin microbiota composition of those who with greater exposure to the natural environment. A study by Hanski et al.^16^ found lower diversity of *gamma-Proteobacteria* on the skin of in homes surrounded by low biodiversity. The amount of forest and farmland surrounding the homes of 6-month-olds in Finland was seen to differentiate the presence of the *Proteobacteria* and *Acinetobacter.*^17^ Cohort studies focusing on the infant gut microbiota associations with the local environment are, however, rare. A systematic review by Van Pee et al.^18^ reported four studies showing positive associations between green spaces and intestinal bacteria richness, evenness, and diversity in adults; however, no significant associations were found in two further studies. A study of early and late infancy from Turku, Finland, revealed no association between residential greenness and the gut microbiota community composition in either early or late infancy, and the degree of human impact and intervention in the local residential area was significant after adjustments.^19^ A study from the CHILD cohort from Edmonton, Canada, revealed that green space proximity and inhalant atopic sensitization at 3 y of age were possibly mediated by intestinal Actinobacteria diversity 4 month.^20^ However, the CHILD cohort reported a reduced odds ratio of alpha diversity when exposed to any natural environment at 4 month in 355 infants.^21^

The environmental microbiota can originate from various sources, but the soil represents the largest reservoir. However, the upper soil is aerobic and favors aerobic bacteria, which are essential for plant life, while the human gut is anaerobic. There is little overlap between the soil and gut microbiota at both high and low taxonomic levels.^22^ The gut microbiota is largely dominated by the Bacteroidetes and Firmicutes phyla, while Proteobacteria and Verrucomicrobia dominated the soil samples. A mouse study has shown changes in the gut microbiota when soil is introduced to cages opposed to clean bedding, with a higher proportion of Bacteroidetes relative to Firmicutes in soil cages.^23^ However, mice would eat soil and are known for coprophagy.

We examined the infant gut microbiota in the first 2 y of life as part of the HELMi (Health and Early Life Microbiota) cohort^24^ in the capital region of Finland and identified associations between gut bacterial compositions and a wide range of environmental variables with adjustment for potentially confounding variables.

## Materials and methods

### Study population and environmental data

This study has received written informed consent from parents/guardians for the use of samples and data from the children used in this study. The study was approved by the ethical committee of The Hospital District of Helsinki and Uusimaa, Finland (263/13/03/03 2015), and performed in accordance with the principles of the Helsinki Declaration.

The HELMi cohort^24^ is a longitudinal birth cohort from the greater Helsinki metropolitan area. Infants were enrolled during the recruitment period between 2016 and 2018. Only healthy singleton infants born term on gestational weeks 37–42 were part of the cohort. Fecal samples were taken at 3 (*N =* 675), 6 (*N =* 682), 13 (*N = 692)*, 26 (*N = 663*), 39 (*N =* 562), 52 (*N =* 655), 78 (*N =* 729) and 104 (*N =* 713) weeks. For this study, we use data from 893 infants (5371 fecal samples) who did not move home during their first 2 y of life and completed metadata surveys.

All environmental variables are defined in the Supplementary Table 1. Outdoor air pollution data were obtained using the SILAM (System for Integrated modeLling of atmospheric coMposition) modeling system,^25^ with a resolution of 0.2 degrees, at the geographic location of the home. SILAM is a global-to-meso-scale dispersion model developed for atmospheric composition, air quality, and emergency decision support applications. The model incorporates both Eulerian and Lagrangian transport routines, eight chemico-physical transformation modules (basic acid chemistry and secondary aerosol formation, ozone formation in the troposphere and the stratosphere, radioactive decay, aerosol dynamics in the air, pollen transformations), three- and four-dimensional variational, and ensemble Kalman filter and smoother data assimilation modules (https://silam.fmi.fi/).

We calculated the 12-month annual averages of the 102 atmospheric constituents after birth (Supplementary Table 2). Due to high collinearity in the air quality data, we performed a principal components analysis (PC) analysis on the SILAM data and utilized the first six PC scores in the models. Nomenclature was based on correlations between the PC scores and 100 atmospheric constituents and was only created for PC scores which were significantly associated with the microbiota in this study.

Biodiversity zonation data describing the biodiversity of Finnish forests^26^ were also utilized to capture the environmental biodiversity at the national level (NAT). Three biodiversity classes were utilized: NAT1, NAT2, and NAT6. NAT 1 is the lowest descriptive version, providing only information on the degree of deadwood, exposing areas with lots of trees, tree species, and rare forest environments; NAT 2 adds penalties for forestry operations, taking human actions into account; NAT6 combines all biodiversity-related information and provides a list of the most valuable forest areas and landscapes.

Land use classification (LUC) was obtained from the Corine Land Cover 2018 at 20 m resolution using level 2 groupings, apart from of ‘Industrial, commercial and transport units’, which was broken down to the level 3 definitions of ‘Road and rail networks and associated land’, ‘Port Areas’, and ‘Airports’. The traffic volume (buffered), proximity to different road types in meters, and length of roads in meters (buffered) were obtained using the digiroad 2018 dataset.^27^ The percentage of LUC within the buffered space was the variable used. There are eight different classifications of road types (freeway, motorway, main road, main street, local road, private road, dirt road, pedestrian/cycle path). We utilized the road types 1 through 4 for total road length within each buffer size, and all eight categories for distance to nearest road by road type.

### Microbiota analysis

Bacterial DNA was extracted from the fecal samples using a previously described bead beating method^28^ (Ambion MagMAX™ Total Nucleic Acid Isolation Kit (Life Technologies)) and KingFisherTM Flex-automated purification system (Thermo Fisher Scientific) as previously described.^29^ 16S rRNA gene amplicon sequencing was performed using Illumina MiSeq and HiSeq platforms for V3-V4 (primers 341 F/785 R) at the Functional Genomics Unit and Institute for Molecular Medicine Finland, University of Helsinki, Helsinki, Finland. The sequencing reads were processed using R package mare,^30^ which relies on USEARCH^31^ for quality filtering, chimera detection, and taxonomic annotation. Forward reads (V3), which were truncated to 150 bases, were used.^32^ Reads occurring <50 times were excluded as potentially erroneous. The taxonomic annotation was performed using USEARCH^31^ by mapping the reads to the Ribosomal Database Project (RDP) taxonomy database version 18,^33^ restricted to known gut-associated taxa. Taxonomic annotations were verified using RDP classifier and in cases of disagreement, the Blast annotation was used. Potential contaminants were filtered by removing reads appearing in negative controls (PCR or extraction blanks) in corresponding numbers from all samples. The sequencing depth cutoff was chosen to be 3000 reads after QC to samples collected at 3 month or before and 5000 paired reads for the remaining samples based on species richness to sequencing depth evaluations.

### Statistical analyzes

The read counts were summarized at the genus level, and the data were transformed into relative abundances by dividing with the total read count. Multivariate analysis of variance was conducted using the adonis2 function in the R package vegan.^34^ For this, the relative abundances were log-transformed and the Pearson correlation distances calculated. Each environmental variable was modeled separately at each time point, adjusting for birth mode and intrapartum antibiotic exposure, diet (breastmilk, formula, mixed, with or without solid foods), number of siblings, pets, maternal education, and type of housing (single-family house, row house, apartment).

The taxon-specific associations were modeled using negative binomial mixed effects models with the data from the first 6 month combined into one model and the data from the subsequent time points (9 month–2 y) into another model. The cohort description at these two groupings can be seen in Table 1. The infant gut microbiota changes drastically during complementary feeding and typically occurs at 6 month of age, making this a good time for grouping the data for ease of summarization. This grouping allows for better summarization of the longitudinal data. All the environmental variables with a significant association to the overall microbiota composition at any time point were combined with the confounding variables into a single model, which was reduced using AIC to identify a significantly associated set of variables for each taxon. For these models, the environmental data were scaled and centered to be able to compare the estimates of variables measured on different scales. All *p*-values reported were corrected for false discovery rate (FDR).

**Table 1.** Description of cohort for both groupings for infants ≤6 month, and (9 month–2 y). The table shows the breakdown for all adjustments used in the models.

	*N* (%) (≤6 month)	*N* (%) (9 month–2 y)
Total children	786	781
Infant sex (male/female)	401/385 (51.0%/49%)	399/382
Pets in home	290 (36.9%)	279 (35.7%)
Birthmode		
Vaginal (No intrapartum antibiotics)	530 (67.4%)	502 (64.3%)
Vaginal (intrapartum antibiotics)	140 (17.8%)	146 (18.7%)
Cesarean section	116 (14.8%)	113 (17.0%)
Number of older siblings in home		
0	382 (48.6%)	390 (50%)
1	308 (39.2%)	309 (39.6%)
2	78 (9.9%)	67 (5.6%)
3+	18 (2.3%)	15 (1.9%)
Type of housing		
Apartment	415 (52.8%)	420 (53.8%)
Single-family house	143 (18.2%)	134 (17.2%)
Row house	228 (29.0%)	227 (29.1%)
Diet		
Exclusively breastfed	305 (38.9%)	0
Formula	6 (0.8%)	0
Breastfed-formula	75 (10.0%)	0
Breastfed-solids	190 (24.2%)	252 (32.4%)
Formula-solids	30 (3.8%)	175 (22.5%)
Breastfed-formula-solids	179 (22.8%)	352 (45.2%)

## Results

The gut microbiota composition was analyzed based on 16S rRNA gene amplicon sequencing and summarized at the genus level. The overall association between the living environment and the gut microbiota was assessed using multivariate analysis of variance, adjusting for multiple background parameters.

The infants lived primarily in urban areas, with mean percentage of LUC for urban fabric just above 50%. This is followed by forests (35.70%), industrial/commercial (13.03%), and road/rail (10.25%) (Supplementary Table 3). Correlations of environmental variables were performed at 750 m, with the majority showing Pearson correlations less than 0.5 (Supplementary Figure 1). The only environmental variables that were correlated more than 0.5 were between Air quality 12 m PC sites 3 – Traffic mean, Industrial/commercial – Road/rail, forests – shrub/herbaceous vegetation.

**Figure 1.**
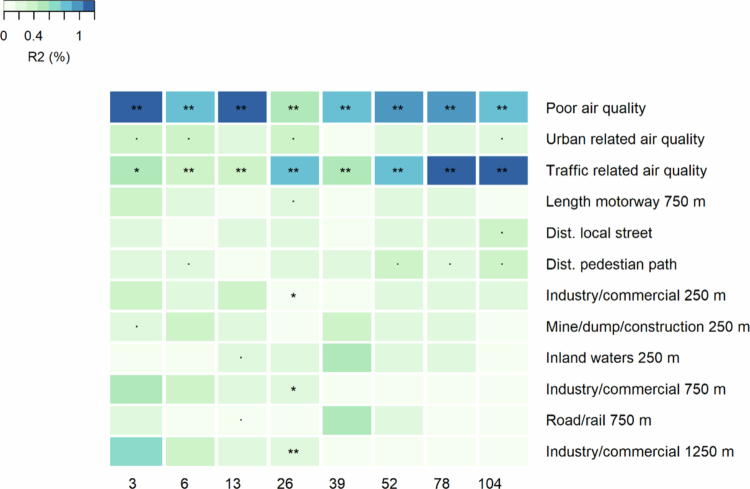
Associations between the gut microbiota at the genus level in the first 2 y and the living environment, based on permutational multivariate ANOVA (adonis) after adjusting for birth mode and intrapartum antibiotic exposure, diet (breastmilk, formula, mixed, with or without solid foods), number of siblings, pets, maternal education, and type of housing (single-family house, row house, apartment). The variance explained (%) is indicated by the color and the asterisks indicate the FDR-corrected *p*-value (*p* < 0.1; *, *p* < 0.05; **, *p* < 0.01; ***, *p* < 0.001). Only significant variables are shown.

After the adjustments, air quality variables were significant throughout the first 2 y of life, with overall poor air quality and traffic-related air quality being the most pervasively associated with the gut microbiota at the genus level (Figure 1; Supplementary Table 4). The maximum amount of the variance explained by any of the environmental variables was 1.49% (traffic-related air quality at 2 y). Industry and commercial infrastructure at all buffer sizes were significant at 26 weeks.

**Figure 2.**
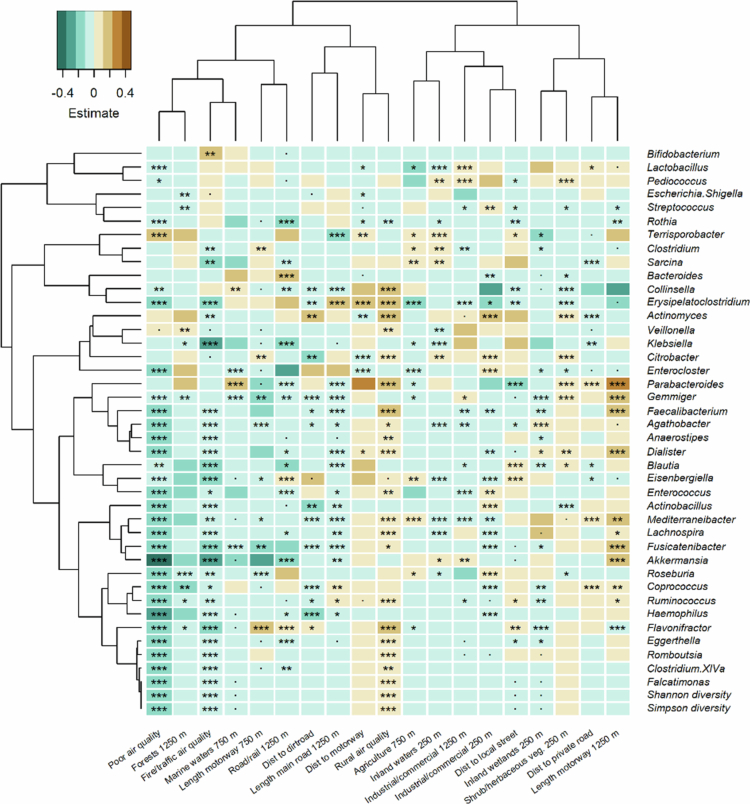
Associations between individual gut bacterial genera in the first 6 month of life (*N* = 2712) and the living environment, based on generalized linear models with a binomial distribution after adjusting for birth mode and intrapartum antibiotic exposure, diet (breastmilk, formula, mixed, with or without solid foods), number of siblings, pets, maternal education, and type of housing (single-family house, row house, apartment) and random effects. The model estimate (beta) is indicated by the color and the asterisks indicate the FDR-corrected *p* value (*p* < 0.1; *, *p* < 0.05; **, *p* < 0.01; ***, *p* < 0.001). Only significant variables are shown. All the environmental variables from the final model are shown.

During the first 6 month (Figure 2; Supplementary Table 5), overall poor air quality (Supplementary Table 1 for variable descriptions), was overall negatively associated with multiple bacterial genera, while inland wetlands within 250 m was only positively associated with *Mediterraneibacter.* Both Shannon and Simpson diversity were significantly negatively associated in the presence of poor air quality in the first 6 month.

**Figure 3.**
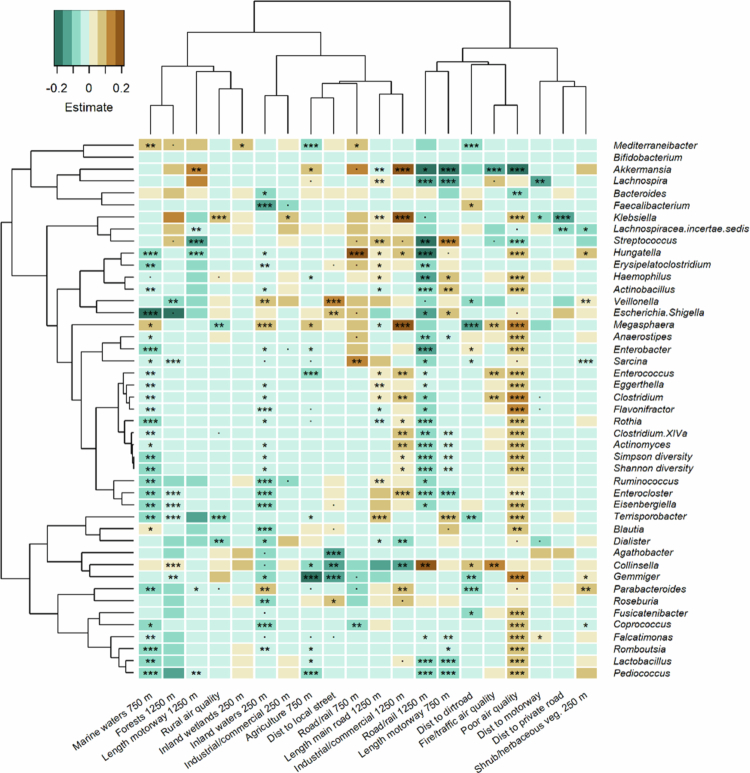
Associations between individual bacterial genera at 9 to 24 month (*N* = 2659) and the living environment, based on generalized linear models with the binomial distribution after adjusting for birth mode and intrapartum antibiotic exposure, diet (breastmilk, formula, mixed, with or without solid foods), number of siblings, pets, maternal education, and type of housing (single-family house, row house, apartment) and random effects. The model estimate (beta) is indicated by the color and the asterisks indicate the FDR-corrected *p* value (*p* < 0.1; *, *p* < 0.05; **, *p* < 0.01; ***, *p* < 0.001). All the environmental variables from the final model are shown.

Between 9 and 24 month (Figure 3; Supplementary Table 5), poor air quality had the strongest association with the infant gut microbiota composition with significant positive associations with a range of genera, while negatively associated with *Akkermansia*. Forests (1250 m), agricultural areas (750 m), and marine waters (750 m) were negatively associated with several bacteria genera. Forests were negatively associated with *Rothia*, *Lactobacillus*, *Lachnospira*, and *Hungatella*, while agriculture with Faecalibacterium, *Enterocloster*, *Blautia*, and *Ruminococcus* and marine waters with *Pediococcues*, *Gemminger*, and *Collinsella*. The Shannon and Simpson diversities were significantly positively associated with overall poor air quality.

## Discussion

Utilizing a longitudinal cohort of 893 term-born infants followed from birth to the age of 2 y who maintained a single place of residence throughout, we delineated the general associations between the gut microbiota in the first 2 y of life and environmental exposures. Our results show that, after adjusting for important confounders, the surrounding environment has a weak but significant association with the infant gut microbiota composition near the home. Air quality explained the largest degree of variance of all environmental variables and were associated with the largest number of genera in the infant gut. Only industry/commercial LUC had any significant association with microbial composition in the first 2 y, while environmental LUCs, such as forests, agriculture, marine waters and inland wetlands near the home were associated with several genera. These results highlight that it is not the environment, but rather important life history events such as birthmode, lifestyle and medical practices, determine the microbiota composition in early life. Additionally, the lack of significance of the environment in explaining the gut microbiota suggests that it is not a key source of microbes as proposed by the Biodiversity hypothesis^35^ for the infant gut. Focus should therefore be placed on improving practices and lifestyle choices which promote the health and development of infants in early life. As the environment is not a reservoir for the anerobic bacteria which inhabit the gut, any influence that the surrounding natural environment would have on the gut microbiota would likely be from exposure to pollutants through air or water.

Studies that attempt to observe and quantify the impact of the environment on the gut microbiota often lack appropriate confounders, such as diet, ethnic/cultural differences, family history and medical background.^15^^,^^36^^,^^37^ The proximity to forests or forest-related biodiversity near the home, which represent the largest natural environment in Finland covering 67% of the total land area, were not significantly related to the gut microbial composition during the first 2 y and only associated with any a small number of genera after 6 month of age. The maximum amount of the variance explained by any of the environmental variables was only 1.49%, while we have previously shown with the same data that infant and family-related variables together explain up to 30% of the variance.^8^ Thus, in comparison, the impact of the outside environment on infant gut microbiota is small.

Air quality was significantly associated with gut microbiota variation throughout the first 2 y. Exposure to NO_2_, but not residential green spaces, has been associated with a reduction in alpha diversity, as well as changes to the infant gut microbial composition during the first year of life.^38^ O_3_ exposure has been associated with decreased microbial diversity and a higher abundance of *Bacteroides* spp. in the infant gut.^39^ Our study found that diversity was negatively associated with poor aur quality in the first 6 month, although this trend reversed after that Bailey et al.^40^ saw positive associations between particulate matter with a diameter 10 microns or less (PM_10_) and the genera *Dialister, Dorea, Acinetobacter*, and *Campylobacter* in the gut microbiota of infants at the age of 6 month were similarly associated in this study. Roads and traffic are known contributors to PM that are inhalable into the lungs and can induce adverse health effects. It has been put forward that air pollution may influence the gut microbiota through mechanisms such as gut epithelial damage and permeability, inflammation, and oxidative stress.^41^ This study was not interested in the specific effects of individual pollutants, rather the atmosphere as a whole, on the gut microbiota. As such, we utilized the principal components from the SILAM model to better incorporate this idea. By combining a wide swath of atmospheric constituents (over 100), we were able to better account for the high degree of collinearity which exists between common atmospheric pollutants.^42^

Studies on the impact of local environment on the gut microbiota have shown contrasting results. Cross-sectional studies on infants and adults saw that increasing residential greenness levels were associated with a corresponding decrease in gut microbiota diversity.^21^^,^^43^ However, when comparing residential greenness using vegetation indices from 34 countries and the gut microbiota of adults, an increase in microbial diversity was observed as well as higher relative abundance of *Bifidobacterium* and lower abundance of *Holdemania, Anaerotruncus,* and *Streptococcus.*^44^ Whether this was in fact mediated by improved local air quality, is not clear, since the study did not adjust for air quality.

Agriculture and forests near the home had significant negative associations with several genrea after 6 month, Modern agriculture practices utilize a variety of pollutants such as pesticide and fertilizers, and there is evidence that people living near fields have an increased exposure than the general population.^45^ *In-vitro* studies found decreased abundance of *Bifidobacterium* and *Lactobacillus* and increases of *Enterococcus* and *Bacteroides* when exposed to chlorpyrifos (CPF),^46-48^ the most widely used organophosphate pesticide.^49^ We observed a decrease in *Ruminococcus*, *Blautia*, and *Enterocloster* after 6 month.

It was recently reported that infants born during very strict pandemic restrictions, during which infants had very limited exposure to outside environments, had a benign gut microbiota development with a high abundance of bifidobacteria and fewer than expected cases of atopic dermatitis,^50^ demonstrating that the infant gut microbiota is not strongly dependent on environmental exposure. While several genera have significant association the environmental variables in this study, only four LUCs (inland wetlands, forests, marine waters and agriculture) were significant. No significant associations were found between the biodiversity indices and the infant gut microbiota. The degree to which the environment influences the gut microbiota as found in the present study corroborates the findings of Korpela et al.^50^ on the overall independence of the gut from the environmental microbial world. Exposure to the environment seems to influence the gut microbiota mostly indirectly. This is possibly through pollution affecting the host's immune system rather than directly introducing colonizing microbes.^51^ Maternal exposure to air pollution during pregnancy and during lactation has been seen to affect infants after birth and the composition of human milk oligosacrides.^52^ Colonization of environmental microbes would also be difficult as the human stomach has a pH ranging from 1.5 to 3.5, although newborns have a less acidic stomach. Studies have shown that pH < 4 results in the killing of 99.9% of bacteria within about 90 min,^53^ limiting the potential of microbes on food or from the environment to colonize the gut. It is thus not clear how environmental microbes would plausibly influence the human gut microbiota. Environmental pollution (e.g. PM_2.5_, volatile organic compounds found from indoor gas appliances and exposure to agricultural biproducts, such as pesticides, endotoxins, and allergens) has been shown to upset the immune balance similarly to viral infections.^54-57^ The results suggest that the impact of urbanization on gut microbiota may be partly driven by increased exposure to pollution, rather than by limited exposure to environmental microbial diversity.^54-57^

While this study has several strengths in terms of a large number of subjects, longitudinal sampling, and a variety of environmental exposure and confounder variables it is limited in that it uses 16 s rRNA data. 16 s rRNA limits taxonomic resolution to the genus level and does not allow for functional or species and strain-level taxonomic insights. This could be rectified through the use of metagenomic sequencing. Ongoing research is focused on improving the understanding of factors associated with the health and development in early life with regards to the infant gut microbiota.

## Supplementary Material

Supplementary material Supplementary Table 5_v2

Supplementary material Supplementary Table 2

Supplementary material Supplementary Table 4

Supplementary material Supplementary Table 3

Supplementary material Supplementary Table 1

Supplementary material Supplementary Figure 1

## Data Availability

The HELML microbiome 16S rRNA gene sequences in this study have been deposited in the European Nucleotide Archive (ENA) under accession code (https://www.ebi.ac.uk/ena/browser/view/PRJEB55243), along with limited metadata (collection date, sex, age in weeks, geographic location, and sequencing method). Additional individual-level metadata, even pseudonymized, are sensitive and are protected by the GDPR and not publicly available. Reasonable data sharing requests based on data processing and material transfer agreements can be made to Anne Salonen, University of Helsinki, Finland. (anne.salonen@helsinki.fi).
